# Case of Vaccine Vesicle Occurring Six Months after the Insertion of the Virus

**Published:** 1823-02

**Authors:** W. Baker


ART. IX.—*Case of Vaccine Vesicle occurring six Months after the insertion of the Virus.* By W. BAKER, M.D.

SOME short time ago, I was requested by a lady to examine a cow-pox vesicle on her child's arm, a fine boy, about eighteen months old. I found the pustule of good size, well formed, the areola completed, in every way presenting the satisfactory appearances we expect to find about the tenth or eleventh day after insertion. The history of the case, as I received it from the mother, (and which I found, from more minute after inquiry, to be in every way correct,) is as follows:

Rather more than six months before, the child had been vaccinated in the site of the present pustule, but, no infection taking place, fresh matter was inserted into the same arm about eight or ten days afterwards, rather lower than the spot first inoculated. The second inoculation communicated the disease in the usual time; and I am assured the child showed the usual trifling symptoms of indisposition, and the pustule presented every satisfactory appearance. The cicatrices of both pocks now remain, and are equally well defined.

From this child having experienced secondary cow-pox, it will most probably be argued the first was spurious and inefficient; as I believe it is considered, by most practitioners, we cannot excite the genuine pustule a second time. It is true, I cannot vouch for the first from my own observations; but, from the parents, I am assured it was in every way deemed satisfactory; and that, when at the height, the swelling and inflammation which attended were equal in extent to what I witnessed in the second.

From the pathologist, this undoubtedly will claim some share of attention; and, though I may fail, from this solitary instance, to establish it as a proof that the same system may be twice placed under the full influence of the vaccine disease, it must still be considered as rather a curious anomaly. In the second disease, I think I am justified in saying the child was fully under the influence of the disease; for, though it is almost impossible, particularly in an infant, to define the extent to which the system might participate, in the usually mild form in which the disease presents itself, yet the satisfactory appearance of the pustule, and the usually attendant characteristic inflammation, bespeak such local susceptibility to specific action, that I think I am entitled to say there was an universal consent of the system.

I presume every virus has its own peculiar laws, observing some definite periods in its development and termination, and a similar order in the succession of its intermediate symptoms. Anomalies will occasionally present: they may be considered modifications consequent upon local or systematic peculiarities, but the real generic characters of the disease, by a nice discriminator, will probably still be found. Hydrophobia is universally admitted to be the most irregular in its periods of attack; unfortunately for mankind, it has hitherto observed a greater uniformity in its termination. In a former publication of mine, (Review of the Medical Treatment of Hydrophobia,) I referred to several well-authenticated cases occurring six and nine months, and two years, after communication with a rabid animal; and, beyond those periods, several cases are on record.

This little case of variola vaccina I should not have considered worthy a place in your scientific Journal, as a mere proof of recurrence of disease, excited by successive inoculations, and appearing in the usual periods after insertion. It is the irregular succession in this case, but most particularly on account of the length of time (about six months,) which elapsed betwixt the second disease and the first inoculation, from which it unquestionably took its rise, that I have deemed it worthy the attention of the profession.

*Howden; October* 1822.

